# Meeting Report: Fungal Genomics Meets Social Media: Highlights of the 28th Fungal Genetics Conference at Asilomar

**DOI:** 10.1534/g3.115.024158

**Published:** 2015-12-01

**Authors:** Michelle Momany, Antonio Di Pietro, William G. Alexander, Bridget M. Barker, Omar S. Harb, Sophien Kamoun, Francis Martin, J. Chris Pires, Jason E Stajich, Bart P. H. J. Thomma, Sarah Unruh

**Affiliations:** * Department of Plant Biology, University of Georgia, Athens, Georgia 30602; † Department of Genetics, University of Cordoba, Cordoba, Spain 14071; ‡ Laboratory of Genetics/DoE Great Lakes Bioenergy Research Center, University of Wisconsin-Madison, Wisconsin 53706-1580; § TGen-North, Flagstaff, Arizona 86001; ** The Eukaryotic Pathogen Databases, Department of Biology, University of Pennsylvania, Philadelphia, Pennsylvania 19104-6021; †† The Sainsbury Laboratory, Norwich Research Park, Norwich, United Kingdom NR4 7UH; ‡‡ Laboratoire d’excellence ARBRE, UMR INRA-Université de Lorraine ‘Interactions Arbre/Microorganismes’, Champenoux, France 54280; §§ Division of Biological Sciences, University of Missouri-Columbia, Missouri 65211; *** Department of Plant Pathology & Microbiology, University of California, Riverside, California 92521; ††† Laboratory of Phytopathology, Wageningen University, Wageningen, The Netherlands 6708 WG

The 28th Fungal Genetics Conference was held March 17−22, 2015, at the Asilomar Conference Center in Pacific Grove, California (http://www.genetics-gsa.org/fungal/2015/index.shtml). Arguably the most popular of international fungal genetics conferences, the Asilomar meeting reached its registration cap 2 days before the early bird deadline, with 910 participants from 35 countries.

One striking feature of this year’s meeting was the high level of Twitter participation. On the basis of analytics from the health care social media analytics company Symplur (http://www.symplur.com/healthcare-hashtags) the hashtag #Fungal15 racked up 3456 tweets from 349 participants, and tweets were seen by more than 3 million others.

As is traditional, the meeting co-organizers have been asked to summarize highlights of the conference. As is also traditional, such a summary is a nearly impossible task with 20 plenary talks, 216 concurrent talks, and 662 posters to be considered. In recognition of the high level of social media participation and to give greater coverage, scientific co-chairs Michelle Momany (University of Georgia) and Antonio Di Pietro (University of Cordoba, Spain) invited the top tweeters to join us in picking highlights of the 28th Fungal Genetics Conference. Even so, these highlights were not able to cover all the terrific science at the meeting.

## Mycotweets

Perhaps not surprisingly, several of the top tweeters mentioned participation in the twitter subculture (along with the Genetics Society of America’s support of and participation in social media) as a meeting highlight, noting that active use of Twitter during the meeting actually provided an additional venue for scientific discussion and sharing of ideas. This type of access was especially useful during concurrent sessions, allowing followers of #Fungal15 to get a taste of talks they were unable to attend. Twitter also was the subject of a talk in the education and outreach session from Cat Adams (University of California Berkeley), who pointed out that academic tweets are nine times more likely to be rebroadcast and that 30% of tweets sent by academics contain a link to a peer-reviewed resource. Because Twitter can be intimidating to new users, Adams thoughtfully provided newbie tips for effective communication (available for public download at http://www.scienceismetal.com). The education and outreach session also featured a number of talks that highlighted the use of fungi as tools in undergraduate teaching and useful online resources, including the Genetics Society of America peer-reviewed education portal (http://www.genetics-gsa.org/education/).

## Fungal Genetics Is Now Fungal Genomics

Virtually every area of research incorporated an “-omics” component. Although next-generation sequencing has allowed model fungi to push forward, its benefits have been especially notable for less-developed systems. One outstanding example of genomics advancing a complex biological system came from Rachel Dutton (Harvard University), whose group is studying patterns of microbial diversity in the surface-associated communities that make up the rind of major types of cheeses—edible biofilms. High-throughput sequencing of 137 different rind communities across 10 cheese-loving countries revealed 24 widely distributed and culturable genera of bacteria and fungi as dominant community members. Intriguingly, highly reproducible community types evolved independent of geographic location of production. Microbial communities of cheese rinds and other fermented foods represent an experimentally tractable system for defining ecological and genetic mechanisms that influence microbial community assembly and function.

Dutton’s talk also inspired one of the more unique hashtags of the meeting #96wellcheeseplate; from Ken Bruno @KSBruno9

Several presentations focused on genome structure, evolution, and links to epigenetics. Toni Gabaldón (Centre of Genome Regulation, Barcelona, Spain) postulated that the *Saccharomyces* whole-genome duplication arose after hybridization of two species rather than genome doubling of a single species. He presented compelling evidence for the existence of a coetaneous interspecies hybridization, providing a new perspective for interpreting the origin and consequences of genome expansions.

High-quality genome assemblies in *Leptosphaeria maculans* (Thierry Rouxel, INRA-BIOGER, Grignon, France), *Zymoseptoria tritici* (Daniel Croll, ETH Zurich, Switzerland; Jessica Soyer, University of Kiel, Germany), and *Verticillium dahliae* (Michael Seidl, Wageningen University, Netherlands) provided new tools for analyzing sequence and structural polymorphisms and their links to pathogenicity in populations of these species. For example, comparative analysis of *V. dahliae* genomes revealed recent segmental duplications that established lineage-specific regions, suggesting that evolution is linked to segmental genome duplications and rearrangements mediated by improperly repaired DNA breaks.

Although epigenetic marks have been poorly characterized in filamentous fungi outside the model species *Neurospora crassa*, the role of epigenetics in pathogenicity is actively being investigated. So far, it appears that repeat-rich areas show increased marks of methylation. Mark Gijzen (Agriculture and Agri-Food Canada) reported on effector gene silencing in *Phytophthora.* Intriguingly, his results suggest strain-specific epigenetic modifications enable the pathogen to evade plant immune recognition while the effector gene itself is maintained.

## New Tools and Resources

A highlight throughout the meeting was the sharing of new tools and techniques in a variety of fungal systems. Many concurrent sessions were organized around these themes: “Fungal Biotechnology” focused on mining fungi for enzymes; “Environmental Metagenomics” showed us how next-generation sequencing technologies are providing insight to the microbial world beyond just bacteria; “*In vivo* Imaging of Host-pathogen Interactions” illustrated what technologies are available to observe these interactions in real time for both plant and animal pathogens (#zebrafish); some great tools and ideas were shared in the education and outreach session, particularly with respect to navigating social media; and finally the “Synthetic Biology” session featured probably one of the hottest new techniques—the CRISPR/cas9 system as presented by Christina Noedvig (Technical University of Denmark, The Netherlands), which was #standingroomonly—and highlighted our ability to advance fungal biology in new and exciting ways.

## Celebration of Fungal Diversity: Not Just the Usual Suspects

Many talks and presentations presented a perspective on fungal biology beyond the model filamentous and yeast systems. In her plenary session talk, Emily Troemel (University of California, Davis, USA) described her laboratory’s work on Microsporida parasites of worms. Her studies are building an understanding of the biology of Microsporidia through genomic comparisons, identifying unique patterns in gene families like hexokinases that may be important in host cell reprogramming. The “Extremophilic Fungi” concurrent session offered a plethora of speakers covering fungi that excel in hot, arid, or salty environments. For example, Nina Gunde-Cimerman (University of Ljubljana, Slovenia) reported on a group of halotolerant and human pathogenic fungi, such as *Aureobasidium pullulans*, that thrive in a variety of extreme environments and most surprisingly are commonly found in dishwashers.

The “Early Diverging Fungi” session included many talks that highlighted how genomic technologies are providing new looks at these enigmatic species. Mary Berbee (University of British Columbia, Canada) presented a novel hypothesis about the evolution of pectinases in chytrid fungi and the implicated role in the metabolism and trophism of these fungi. Alisha Quandt (University of Michigan) introduced work using single-cell genomics to sequence the genomes of Cryptomycota lineages that are parasites of other fungi and protists. Other notable talks included phylogenomics of the Zygomycetes from Jason Stajich (University of California, Riverside), studies of *Paramicrosporidium saccamoeba* that are parasites of Amoeba nuclei by Alisha Quandt (University of Michigan), and genome analysis of *Nosema cernae*, revealing recent population expansion by Nicolas Coradi (University of Ottawa, Canada). Yet another highlight was the talk by Olga Lastovetsky (Cornell University) on fungal-bacterial symbioses between *Rhizopus* and endosymbiotic *Burkholderia* bacteria, revealing extensive signaling that precedes the establishment of symbiosis. In her plenary talk, Rosa Ruiz-Vazquez (University of Murcia, Spain) presented the mechanisms of RNA silencing in the Zygomycete *Mucor circinelloides*, an opportunistic pathogen of humans, and reported exciting results on the presence of a second Dicer-independent RNAi pathway in the Zygomycetes.

## Evolution Revolution

Many laboratories currently are leveraging a combination of experimental evolution and high-throughput sequencing methods to study the molecular basis of adaptation and the reproducibility of evolutionary outcomes across a variety of model systems. This was elegantly highlighted in a Chair′s Choice talk by Gregory Lang (Lehigh University), who combined the awesome power of yeast genetics with robotics, subjecting hundreds of isogenic *Saccharomyces cerevisiae* populations to laboratory evolution. Taking samples of the populations for analysis via flow cytometry, high-throughput sequencing, and freezer storage (creating an artificial “fossil record”), his group showed that patterns of sequence evolution are driven by a balance between chance events and the deterministic action of selection on individual mutations, thus favoring parallel evolutionary solutions in replicate populations. As sequencing technology continues to improve and sequencing cost to decrease, we are likely to see a striking increase in our ability to test fundamental questions in evolutionary biology of additional fungal model systems, such as *N. crassa*, in the laboratory.

Can’t help feeling that the talk from the Lang lab should have had standing ovations at #Fungal15 Awesome science! Mikael R. Andersen @MRoerdam

## Cell Biology, Cell Cycle, and Fungal Interactions

Several plenary talks highlighted the cytoskeleton and cell cycle. Samara Reck-Peterson (Harvard) gave a beautiful overview of microtubule-based intracellular transport, a fundamental process in all eukaryotic cells. Transport of the molecular motor dynein by a kinesin to the start of the microtubule plus end was shown by the use of *in vitro* reconstitution. She illustrated how the construction of 3D artificial cargos helps to dissect the mechanism of bi-directional, microtubule-based transport. Berl Oakley (Kansas State University) showed that microtubule nucleator gamma tubulin has roles controlling the cell cycle, likely through localization of spindle assembly checkpoint and other regulatory proteins. In an elegant dissection of appressorium development in the rice blast fungus *Magnaporthe oryzae*, Nick Talbot (University of Exeter, UK) showed that appresorium formation is controlled by the cell cycle. By recreating classic cell-cycle mutations, his group demonstrated that appresorium morphogenesis requires passage through the G2/M transition and the DNA replication checkpoint. Nicholas Buchler (Duke University) presented a thought-provoking analysis of the evolution of cell-cycle control in eukaryotes, showing that the SBF transcription factor, a major regulator of the G1/S transition, was likely introduced into fungi by horizontal gene transfer from a DNA virus.

Andre Fleissner (Braunschweig Technical University, Germany) and Louise Glass (University of California, Berkeley) provided two nicely complementary plenary talks on the mechanisms underlying cell−cell communication and hyphal fusion in *N. crassa.* Fleissner showed that the composition of the plasma membrane is critical for proper cell−cell interaction and that mutants accumulating specific ergosterol precursors exhibit defects in the fusion program. Glass showed that self and nonself interactions during germling fusion are linked, that an interplay between these two systems is important in the regulation of chemotropic interactions and cell fusion, and that genes involved in cell fusion are highly polymorphic.

## Novel Roles for Secreted Pathogen Effectors

Several talks introduced new functions for effectors of filamentous pathogens. Bart Thomma (Wageningen University, Netherlands) and Tim Friesen (USDA-ARS and North Dakota State University) showed that LysM effectors are produced by various plant pathogens to protect them against host chitinases and suppress chitin-triggered immunity. Pietro Spanu (Imperial College, UK) presented a novel class of effector proteins in cereal powdery mildews named *R*N*A*se-*L*ike *P*roteins in *H*austoria (RALPH), whose structure is highly similar to that of microbial RNAses and which interact with nucleic acids and with host proteins. Yet another type of secreted virulence effector was introduced by Antonio Di Pietro, (University of Cordoba, Spain): *F*usarium *R*apid *AL*kalinization *F*actor (*f-ralf*) was likely horizontally acquired from plants and affects local pH during infection to promote host colonization. Yasin Dagdas (The Sainsbury Lab, UK) and Elodie Gaulin (University Paul Sabatier, France) reported on functional analyses of oomycete effectors that interfere with defense responses, either by targeting autophagosomes or by triggering host DNA damage in plant and animal cells. Gero Steinberg (University of Exeter, UK) presented a spectacular Chair’s Choice talk on long-distance endosome trafficking that drives fungal effector production, whereas Alga Zuccaro’s (University of Cologne, Germany) plenary lecture provided novel insights into the molecular mechanisms driving endophytic interactions in plants, obtained from genomic studies in sebacinoid root endophytes.

The ways in which effectors of oomycete and fungal pathogens translocate into plant cells remains an unsolved mystery. Stephan Wawra (University of Cologne, Germany) proposed that the Arg-Xaa-Leu-Arg (RXLR) translocation motif of *Phytophthora* acts as a haustorial targeting domain that is cleaved and *N*-acetylated before effector secretion, similar to a related motif in host-translocated effectors of plasmodia. This evokes a model in which RXLR is involved in pre-secretory sorting rather than host translocation *per se*. However, the topic remains controversial since Qunqing Wang (Oregon State University) reported experimental evidence arguing against effector cleavage during infection. Future breakthroughs in this field may originate from new host-translocation assays. Libera Lo Presti (MPI Marburg, Germany) reported promising progress on a BirA biotin ligase based assay of pathogen-dependent effector translocation.

In the closing Perkins/Metzenberg Lecture, Michael Hynes (University of Melbourne, Australia) gave a sharp, entertaining, and highly personal view of his scientific career, including sound advice to early career scientists: read, think, talk to your peers; be generous with your ideas and time; be realistic, flexible and do not neglect your work-family life balance.

**Author Twitter Handles**

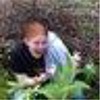
**@sau916**

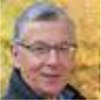
**@fmartin1954**

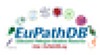
**@eupathdb**

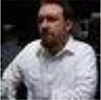
**@mycomancy**

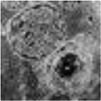
**@drvalleyfever**

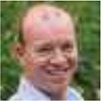
**@team_thomma**

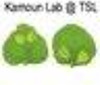
**@kamounlab**

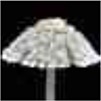
**@hyphaltip**

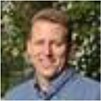
**@jchrispires**

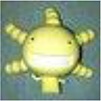
**@mcmomany**

**FGC 28 Welcome Remarks**

We are all here because at some time in the past we chose to work on fungi or organisms with fungal lifestyles. Some of us chose fungi because they are exquisite models. We wanted to answer fundamental biological questions using the systems that gave the world one gene-one enzyme and gamma tubulin, that helped to unravel the control of cell cycle and circadian rhythms.

Some chose fungi because of their massive potential for damage and a desire to stop it. Every year fungal disease kills as many people as malaria or tuberculosis. Perhaps even more stunning, every year 20% of agricultural crops are lost with most of that loss caused by fungi. That lost food is especially important, given our ever-growing population.

Some chose fungi not because of their destructive potential, but because of their great capacity to make and secrete products, a capacity that is a direct result of the fungal hallmark of extracellular digestion. Every year industry uses 1.2 million tons of citric acid in products such as soft drinks. Those 1.2 million tons are made by a fungus. And, of course, fungi were using plants for biofuel long before it was trendy.

Some did not choose fungi for their power as models or their ability to destroy or to make products. They chose fungi for their fascinating and unique biology. Members of the fungal kingdom range from microscopic unicellular yeasts to macroscopic basidiomycetes with mycelia that cover acres. What drives the evolution of such diversity? What drives interactions with the environment and other organisms? With 500 sequenced genomes and hundreds more in the pipeline we can answer such questions at an unprecedented level of detail, a level not even imagined just ten years ago.

Regardless of why you are here, we welcome you and sincerely hope that you will be amazed and inspired at this 28th Fungal Genetics Conference.

-Michelle Momany, Scientific Co-Chair

